# Barriers to and Facilitators of Artificial Intelligence Adoption in Health Care: Scoping Review

**DOI:** 10.2196/48633

**Published:** 2024-08-29

**Authors:** Masooma Hassan, Andre Kushniruk, Elizabeth Borycki

**Affiliations:** 1 Department of Health Information Science University of Victoria Victoria, BC Canada

**Keywords:** artificial intelligence, governance, health information systems, artificial intelligence adoption, system implementation, health care organizations, health services, mobile phone

## Abstract

**Background:**

Artificial intelligence (AI) use cases in health care are on the rise, with the potential to improve operational efficiency and care outcomes. However, the translation of AI into practical, everyday use has been limited, as its effectiveness relies on successful implementation and adoption by clinicians, patients, and other health care stakeholders.

**Objective:**

As adoption is a key factor in the successful proliferation of an innovation, this scoping review aimed at presenting an overview of the barriers to and facilitators of AI adoption in health care.

**Methods:**

A scoping review was conducted using the guidance provided by the Joanna Briggs Institute and the framework proposed by Arksey and O’Malley. MEDLINE, IEEE Xplore, and ScienceDirect databases were searched to identify publications in English that reported on the barriers to or facilitators of AI adoption in health care. This review focused on articles published between January 2011 and December 2023. The review did not have any limitations regarding the health care setting (hospital or community) or the population (patients, clinicians, physicians, or health care administrators). A thematic analysis was conducted on the selected articles to map factors associated with the barriers to and facilitators of AI adoption in health care.

**Results:**

A total of 2514 articles were identified in the initial search. After title and abstract reviews, 50 (1.99%) articles were included in the final analysis. These articles were reviewed for the barriers to and facilitators of AI adoption in health care. Most articles were empirical studies, literature reviews, reports, and thought articles. Approximately 18 categories of barriers and facilitators were identified. These were organized sequentially to provide considerations for AI development, implementation, and the overall structure needed to facilitate adoption.

**Conclusions:**

The literature review revealed that trust is a significant catalyst of adoption, and it was found to be impacted by several barriers identified in this review. A governance structure can be a key facilitator, among others, in ensuring all the elements identified as barriers are addressed appropriately. The findings demonstrate that the implementation of AI in health care is still, in many ways, dependent on the establishment of regulatory and legal frameworks. Further research into a combination of governance and implementation frameworks, models, or theories to enhance trust that would specifically enable adoption is needed to provide the necessary guidance to those translating AI research into practice. Future research could also be expanded to include attempts at understanding patients’ perspectives on complex, high-risk AI use cases and how the use of AI applications affects clinical practice and patient care, including sociotechnical considerations, as more algorithms are implemented in actual clinical environments.

## Introduction

### Background

The onset of the 2020 COVID-19 pandemic has particularly triggered health care organizations across the globe to consider transforming their health delivery models. According to the 2024 Global Health Care Sector Outlook report published by Deloitte, hospitals and health care organizations are addressing challenges by turning toward novel technologies such as cloud computing, artificial intelligence (AI), 5G telecommunications, and interoperable data and analytics to enable care via digital models [1]. It was not too long ago, in 2017, when the Canadian government created the Pan-Canadian Artificial Intelligence Strategy and announced an investment plan of CAD $125 million (US $96 million) for research in AI. The Canadian Institute for Advanced Research was mandated to lead this strategy forward with a 5-year plan to enhance Canada’s AI innovation profile on the international stage. Health care is one of the 4 sectors on which the Canadian Institute for Advanced Research is focusing for the advancement of AI research [2]. However, health care has seen slow success in the implementation of AI use cases.

### Objective

The objective of this review was to investigate what is known from existing literature about the barriers to and facilitators of AI adoption in health care and propose recommendations on approaches that would address barriers to adoption.

We begin this paper by (1) defining AI (before providing some context for AI’s use in health care), (2) describing the most prominent applications of AI in health care, (3) outlining the value that AI is expected to provide, and (4) providing a rationale for this review.

### History and Definitions

AI is not necessarily a new concept; rather, the exploration of this innovation goes as far back as 10th century China, when mechanical engineer Yan Shi presented to Emperor Zhou mechanical men capable of independently moving their bodies. In the 12th century, al Jazari, who was a polymath, an inventor, and a mechanical engineer, developed humanoid robots. Furthermore, in the 15th century European Renaissance, Leonardo da Vinci similarly developed a knight robot that was able to move different parts of its body on its own. The definition of AI has changed over time, from referring to robotic machines to much more sophisticated technologies capable of mimicking human decision-making processes and behaviors. The advancement of computer systems and languages in the more recent decades has made it possible to progress toward AI systems. The definition that most fits today’s application of AI and is referenced in this paper was coined by John McCarthy in 1956. McCarthy defined AI as “the science and engineering of making intelligent machines” [3]. It is unclear what definition is consistently used; however, what is clear is that today’s AI encompasses various techniques aimed at mimicking humanlike intelligence and behavior to allow for the emergence of intelligent technologies capable of problem-solving and decision-making. In this way, AI should be skilled at processing large amounts of information, should arrive at a conclusion through reasoning, and have the ability to learn and solve problems on its own [4]. Various analytic techniques are used to allow for this, with the most prominent ones falling under machine learning (ML) and natural language processing (NLP) [5].

Large data sets are needed to develop effective AI algorithms and enable AI’s maturity to arrive at intelligent outputs. In health care, the sources of data for NLP are primarily unstructured data, for example, free-text clinical notes from electronic medical records (EMRs). ML techniques use structured data such as diagnostic images and genomic data. ML uses two primary types of algorithms: (1) supervised and (2) unsupervised. Supervised learning provides more clinically relevant results; hence, AI applications mostly use supervised learning. There are several techniques in supervised learning, with neural networks and support vector machines being the most popular of the techniques [5]. The most modern extension of the neural network technique is called deep learning (DL). DL has been made possible due to the increasing availability of large amounts of complex data. This technique has become more popular because of the number of layers of data it can translate. NLP can be used to convert unstructured data into structured data. Therefore, both NLP and ML, along with additional data, are required to train the AI continuously. The more data that are fed into the AI, the “smarter” it becomes. In health care, data sets can be available from various sources, such as electronic health records (EHRs), laboratory tests, diagnostic imaging, electrodiagnosis, genetic diagnosis, and mass screening [5]. In 2022, the release of ChatGPT (OpenAI) brought to light the power of large language models. This type of chatbot-style generative AI is being considered to enable extracting data from EMRs and converting them into meaningful outputs that can be useful for clinicians by lowering their administrative burden [6].

### Current State of AI Research and Health Care Use Cases

Research in AI has been exponentially increasing, with bibliometric reporting of published articles on the topic of health care having increased at an annual growth rate of 5.12% over the past 28 years. As of 2021, the most significant increases in bibliometric reporting took place in the 3 years before 2021 [7]. According to Tran et al [8], the disciplines with the highest number of publications at the intersection of AI and health include cancer, heart diseases and stroke, ophthalmology, Alzheimer disease, and depression. Most publications on the types of AI used reported on robotics, ML, and DL.

In health care, publications of AI applications are concentrated around operational or administrative efficiency as well as patient care improvement, including better outcomes through improved diagnosis and treatment [9]. AI enhances operational and administrative efficiency by providing administrative support to health professionals and improving performance across the organization. AI can achieve this through, for example, its ability to consolidate and provide the latest and most validated research findings that can support clinicians with up-to-date evidence-based decision-making while providing care and its ability to leverage EHR data to predict data heterogeneity between various hospitals and clinics [7]. Emergency departments are largely found to have successfully applied AI to optimize resource planning and crowd management [10,11]; for example, the Hospital for Sick Children and Humber River Hospital in Ontario, Canada, are using AI to improve emergency department operations by predicting patient surges in the emergency waiting room [12,13].

Use cases aimed at improving care outcomes include predictive analytics around disease outcome prediction or prognosis evaluation and clinical decision support systems [7]. Examples of such cases are found in cardiology and include the early detection of atrial fibrillation via a smartphone-based electrocardiogram or cardiovascular risk assessment via patient records. Other promising areas include neurology, specifically stroke prediction and diagnosis [5]. Gastroenterology AI applications have also been successfully tested, where algorithms are used to predict outcomes in cases of esophageal cancer and metastasis in colorectal cancer [14]. Image-based diagnosis is considered the most successful use of AI applications in health care, largely supporting radiology, dermatology, ophthalmology, and pathology [15]. In a review conducted on the literature on AI use in the emergency department, Kirubarajan et al [11] reported that 50% of the studies found that AI interventions were better able to diagnose various ailments, such as acute cardiac events and hyperkalemia, among other health conditions.

As mentioned earlier, AI requires large amounts of data to learn and apply sophisticated reasoning and accurate problem-solving. In addition to the race toward researching AI use cases, a surge in health care data is further setting the stage to allow for accelerated AI innovations [16]. EMR data; wearable sensor technology; and genomic, pharmaceutical, and research databases offer opportunities to apply AI to the analysis of health data. Approximately 30% of the world’s data volume is generated by the health care industry. The compound annual growth rate of data for health care is expected to reach 36% by 2025 [17]. This growth of data volume in health care is faster than that in manufacturing, financial services, and media and entertainment industries [18]. This sets the stage well for developing AI technologies that can be integrated into health care practice, as algorithms now have more data to provide increasingly sophisticated outputs.

### Contributions of the Research

It is clear that the changing landscape, increasing evidence on AI use cases, and increasing availability of data in health care are setting the path toward realizing real-life applications of AI. However, successful utility requires successful adoption, and a number of studies have reported on the challenges encountered with implementing AI in health care [19-21]. Health care organizations are especially complex and can be resistant to change due to various reasons associated with legacy structures, a shortage of resources, and high demand. An estimated 70% of health IT projects fail [22], and an important characteristic of successful technological implementation is tied to its adoption, which is why adoption is a key component of frameworks such as the unified theory of acceptance and use of technology and nonadoption, abandonment, scale-up, spread, and sustainability theory. These frameworks are used to evaluate and study the acceptance of technologies. For example, the unified theory of acceptance and use of technology framework, which integrates all the available theories about technology adoption, suggests several factors that help understand users’ intention to adopt and use a technology. It looks at all the available theories about technology adoption to evaluate use of information systems [23]. Similarly, the nonadoption, abandonment, scale-up, spread, and sustainability framework has incorporated multiple theories to help study factors influencing “non-adoption, abandonment and challenges to scale-up, spread and sustainability of technology-supported change efforts” [24]. Both emphasize the importance of studying adoption to support the successful uptake of technologies beyond implementation. With these reasons in mind, it is important that organizations understand the barriers to and facilitators of AI adoption to ensure successful AI implementation. In reference to the widely known work of Everett Rogers, famously known as the Rogers diffusion of innovation theory, Cresswell and Sheikh [25] have defined implementation as “the consideration and the introduction of HIT applications,” whereas adoption is defined as “the acceptance and incorporation of HIT applications into everyday practice.”

An initial search was performed to identify whether any consolidated reviews, such as scoping reviews, were already conducted to understand the barriers to and facilitators of AI adoption in health care. During this search, it was found that a majority of the literature seemed to report on a specific area, such as radiology, in a specific setting (hospital or community), and a number of studies were reporting on implementation findings and not necessarily adoption. A few literature reviews on the determinants of and barriers to AI adoption have been conducted, such as the review by Radhakrishnan and Chattopadhyay [26]. However, these reviews span across multiple industries. For health care, 1 systematic review on the *barriers* to AI adoption in health care has been conducted by Assadullah [27]. However, there are no consolidated reviews that consider both the *barriers to and facilitators* of AI adoption in health care at large. Therefore, this review has attempted to explore the latter to provide considerations for health care organizations looking to successfully implement AI technologies via increased adoption.

## Methods

### Overview

This review was guided by the methodology and reporting structure outlined for scoping reviews by the Joanna Briggs Institute as well as Arksey and O’Malley [28]. The stages defined by Arksey and O’Malley [28] were followed to conduct this scoping review: (1) identifying the research question; (2) identifying relevant studies; (3) selecting studies for inclusion; (4) charting the data; and (5) collating, summarizing, and reporting the results

### Stage 1: Identifying the Research Question

Because adoption is a key element of successful cost-benefit realization of technological investments, a general question was formed using the “population, concept, and context” approach [29]. The first component, “population,” included users or potential users of the AI system, such as patients, providers, health care leaders, researchers, and those who were involved with implementing AI systems in various settings. The second component, “concept,” consisted of barriers to and facilitators of any AI technology. The third component, “context,” was centered on barriers to and facilitators of any AI technology in *any health care setting,* leaving this as broad as possible to maintain the paradigms of a true scoping review. A generic question developed was as follows: What are the barriers to and facilitators of AI adoption in health care?

### Stages 2 and 3: Identifying Relevant Studies and Study Selection

In commencing the research, eligibility criteria were defined (as described in the Eligibility section). Once the eligibility criteria were defined, the search strategy was identified, and a search for articles was conducted in the selected databases.

#### Eligibility (Inclusion and Exclusion Criteria)

All published studies and gray literature that reported implementation findings related to adoption or reported factors impacting adoption were considered in this review. Therefore, studies with various designs, including quantitative and qualitative studies, literature reviews, thought articles, conference papers, and reports, were included in the initial search and review. “Health care organizations” were defined as organizations that are engaged in providing care to patients or involved in some aspect of providing agency to health care players. Health care players were defined as anyone involved in the process of providing or receiving care, including policy makers; administrative professionals; clinicians; physicians; and, most importantly, patients and their families. All types of AI technologies were considered in this review. Articles were not excluded based on variations in settings (hospital vs community setting) or countries where the research was conducted. Only articles in English were included. Due to the speed at which the landscape for AI is advancing, only articles that were published between January 2011 and December 2023 (when the search was conducted) were included.

#### Search Strategy

This review is intended to synthesize findings from publications that reported on the barriers to and facilitators of the adoption of AI implementations. A search was conducted on MEDLINE, ScienceDirect, and IEEE Xplore in December 2023. Keywords were selected in reference to the question identified to formulate the scope. Keywords included “artificial intelligence”; “healthcare” or “health care”; “hospital,” “health services,” or “health facilities”; “adoption”; “barriers”; “obstacles”; “challenges”; “facilitators”; and “enablers” (Textbox 1).

**Search query.**

**Query**

- “artificial intelligence” AND healthcare or health care or hospital or health services or health facilities AND adoption AND barriers or obstacles or challenges or facilitators or enablers
- “artificial intelligence” AND health AND adoption AND (Barrier OR Facilitator)

#### Study Selection

A total of 2 reviewers independently screened the articles from the initial search by reviewing their titles and abstracts. Articles meeting the inclusion criteria were identified. Articles that did not meet the inclusion criteria were excluded. Any discrepancies were resolved through discussion.

Of the articles identified, the full text of the semifinal set of articles was reviewed to further refine selected articles. This process was iterative, and some exclusions were made during the writing phase, as the findings evolved. An Excel spreadsheet (Microsoft Corp) was used to record the articles identified. Recordings included the following details: the name of the article, authors, journal, whether the article was peer reviewed, type of paper, discipline, country, region, method, population, end users, and type of AI application (if specified). Duplicate studies were identified and removed to ensure there was no overlap.

### Stages 4 and 5: Charting the Data and Collating, Summarizing, and Reporting the Results

A conventional content analysis approach was used to review the articles, chart the data, and identify themes [30]. Publications meeting the inclusion criteria were reviewed in detail, and an inductive approach was used to identify themes. First, the articles were read in full for the author to immerse into the content. This was followed by carefully reading each article and highlighting key concepts around barriers and facilitators that appeared to repeat across all the articles. These initial key concepts were recorded as themes, and this process helped identify many themes that were further categorized and grouped based on similarity. All data were charted in an Excel table to help with the analysis.

### Ethical Considerations

This work received an ethics exemption from the University of Victoria ethics board due to the nature of research being a literature review.

## Results

### Overview

**Figure 1:**
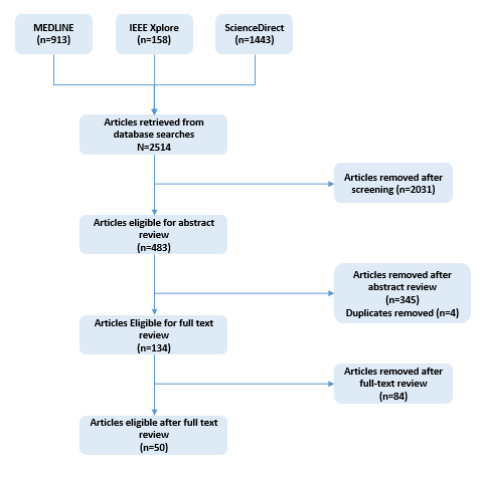
PRISMA (Preferred Reporting Items for Systematic Reviews and Meta-Analyses) flow diagram showing the study selection process.

The initial search from MEDLINE, IEEE Xplore, and ScienceDirect provided cumulative results of 2514 publications. After screening the results, 483 (19.21%) publications were included for abstract review based on the title of the study. After abstract review, 134 (27.7%) publications were identified for further text review, further excluding 345 (71.4%) publications, including 4 (0.8%) duplicate articles. Out of the 134 studies, 50 (37.3%) went through a thorough and more detailed review and thematic analysis. Figure 1 presents the PRISMA (Preferred Reporting Items for Systematic Reviews and Meta-Analyses) flow diagram. The breakdown of studies is based on the country of origin, types of articles selected, and health care discipline or area covered. Overall, 11 articles were from the United States, 7 from China, 5 each from the United Kingdom and Canada, and 3 each from Germany and the Netherlands; the remainder of the articles were from Australia, France, Germany, India, Indonesia and Taiwan, Italy, New Zealand, Saudi Arabia, Singapore, Sweden, Switzerland, and other European countries. In some cases, multiple countries or regions collaborated to publish the articles together, including different European countries or the United Kingdom and United States. A total of 13% of the articles were literature reviews and 8% were mixed methods studies. The rest of the articles were cross-sectional studies, ethnographic or qualitative studies, case studies, white papers, and thought articles. In terms of setting, the majority of the articles discussed AI in health care in general with a majority of the articles reporting from the field of radiology or oncology. The setting of the remainder of the articles were academic hospitals, ophthalmology clinics, hospital, primary care, and dermatology clinics.

### Thematic Analysis

#### Overview

**Figure 2:**
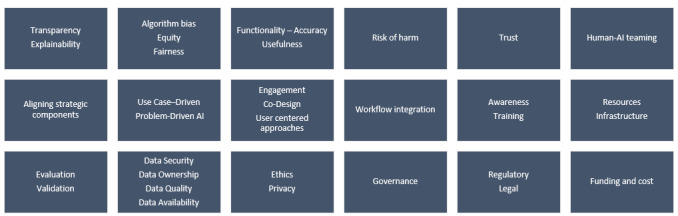
Themes identified. AI: artificial intelligence.

On the basis of the conventional content analysis approach used (as described in the Methods section), a total of 18 categories of barriers and facilitators were identified (Figure 2). Interestingly, the themes were found to provide perspective on both facilitators and barriers. For example, if the theme explainability was identified as a barrier, the same theme was tabulated as a facilitator to capture what the articles recommended for overcoming challenges with explainability to increase adoption. As such, the reporting of the results for each theme provided perspective on the theme being both a barrier and facilitator, with the exception of governance, which was entirely noted as a key facilitator.

#### Transparency and Explainability

Explainability can be defined as the ability to deconstruct an algorithm to understand the mechanism by which it arrived at the output. Explainability has gained prominence due to the fast-paced growth of ML algorithms such as DL. These algorithms are labeled “black box” due to the difficulty in interpreting and tracing the techniques used by the AI models, thereby impacting trust and demanding the need for transparency [31,32]. According to Holzinger et al [33], “explainable AI deals with the implementation of transparency and traceability of statistical black-box machine learning methods, particularly deep learning.” While transparency has a wider definition and explainability is a component of transparency [34], most studies have noted explainability in the context of algorithmic transparency; therefore, findings from these 2 interrelated concepts have been discussed together.

Several studies noted a lack of explainability as a barrier to adoption. Baxter et al [35] reported concerns from adopters around the lack of explainability regarding the prediction of the AI algorithm embedded in the EHR to predict unplanned readmission; specifically, the lack of explainability regarding what features of the algorithm were driving the output was an impediment to trust among adopters. Other studies noted that the way the data were being used to train algorithms was not clear. The lack of traceability and logical understanding of how the algorithm arrived at a recommendation contradicted a key foundation of evidence-based medicine, which relies on high standards of explainability. Clinicians expressed the need to understand both the scientific and clinical bases of the recommendations provided by the AI to confidently validate and apply the decision [19,20,36-38]. Morrison [36] particularly quoted stakeholders seeking clarity on the extent to which there is a need to provide transparency on AI output to patients and how this is directed by legislation or data protection laws. In a study conducted by Nadarzynski et al [39], users of an AI-enabled chatbot reported hesitancy to use the technology due to a lack of transparency on how the chatbot accurately arrives at responses to health inquiries.

According to Holzinger et al [33], “explainability is an important element for consideration in order to enhance trust of medical professionals.” To facilitate adoption, improving algorithmic transparency will be a key consideration to change attitudes and build the trust of adopters [35,40]. Additional recommendations around facilitating adoption were to have processes in place to support clinicians in case of disagreements on decisions due to a lack of transparency and explainability [41]. Furthermore, revealing the process of how the algorithm was developed, who was involved in the development process, whether clinicians were consulted, and how the data were processed would enable acceptability [42,43].

In health care, causality is especially important when using automated decision systems; therefore, Holzinger et al [33] emphasized that AI systems should support the understanding and explanation of the causal models as opposed to simply solving through pattern recognition. Similarly, Gillner [44] also noted that this opacity of the output is not aligned with the “medical ethos.” Weinert et al [45] recommended that investing into explainable AI that produces a transparent and understandable AI could help address the issue of acceptability. Moorman [38] reported that successful adoption was achieved by publishing evidence on the algorithm’s underpinnings and providing clinicians with details on how data elements interacted within the algorithm to produce the predictive output.

#### Algorithm Bias, Equity, or Fairness

A prominent theme that came through was around the prevention of algorithm bias to ensure equity and fairness and avoid concealed discrimination. Algorithmic bias has been defined by Panch et al [46] as “the instances when the application of an algorithm compounds existing inequities in socioeconomic status, race, ethnic background, religion, gender, disability or sexual orientation to amplify them and adversely impact inequities in health systems.” Such biases have been visibly found in glomerular filtration rate and pulmonary function and have continued to persist despite efforts to address them. Inaccurate and underrepresentative training data sets for AI models can cause bias, misleading predictions, adverse events, and large-scale discrimination, causing barriers to adoption [32,41,47,48]. Baxter et al [35] and Chua et al [49] reported clinical stakeholders’ concerns around the relevance of the AI model’s output, especially because the algorithm did not consider social determinants of health to predict risk outcomes for readmissions. Similar concerns were raised by participants from other studies around the risk of algorithm bias as a challenge for adoption. Others expressed dissatisfaction that the AI algorithm may not be representative of the patient population among whom it is implemented or may have been trained with a biased training data set that has been retrofitted to produce certain results, therefore not providing a representative outcome of interest [36,49-51]. AI not accounting for patients’ health determinants was noted as a “grand challenge” [19].

Inadequate data from representative groups, algorithms designed to represent a majority, and missing variables that impact predictions are components that contribute to bias [21]. This can be addressed by engaging clinicians in the design and development of the algorithm to ensure that appropriate measures are taken to address bias before the AI algorithm is deployed.

#### Functionality (Accuracy and Usefulness)

One of the major themes that came through was around the value, usefulness, and accuracy of an AI algorithm. Accuracy and quality of the AI algorithm’s output were primary reasons for adoption hesitancy in the context of the functionality of the AI. In some studies, patients reported the need to assess the usefulness of the AI before using it and had concerns around the quality and accuracy of the output, thereby questioning the value of AI as a whole [39,42,52-55]. Clinicians also reported concerns around the usefulness of the output based on the lack of accuracy and inactionable output contributing to a low likelihood of use. This also included dissatisfaction if recommendations were too similar, inappropriate, or not useful [35,37,38,40,56]. Ease of use; complex interfaces; and inconsistent performance, for example, due to false positives and negatives add burden to the workflow, creating more work for clinicians, thereby impeding adoption [42,52,57-59]. Morrison [36] identified a lack of an agreed standard and benchmark for accuracy (how accurate does an AI tool need to be before it is approved for clinical practice) as an impediment to implementation and, subsequently, adoption, as, if a standard existed, it could provide rationale for the accuracy. Temsah et al [60] recommend the application of evidence-based oversight mechanisms to ensure accuracy and dependability. Finally, Choudhury [61] noted that if an algorithm is not performing up to standards or adds more work to the clinicians, this can impact adoption, as clinicians perceive it as a high risk.

Perceived benefit, perceived usefulness, usability, ease of use, usefulness, accuracy, and reliability of the output of the AI are key contributors to adoption [19,45,62-67]. In particular, usability and acceptability should be assessed with the intended user in mind [41]. Perceived benefit is especially important when it contributes to improved efficiency in clinical processes [62].

From a patient’s perspective, there is value if an AI technology can be used from home for minor consultations, such as skin cancer detection using an AI-enabled app [42]. Ease of use of the technology and accessibility to information for minor health concerns [39] are especially seen as valuable, as they negate the need for a visit to the physician; however, in the event that a visit is required based on the AI’s recommendation, then the integration of the technology with the health care system is considered beneficial [42]. It is essential that as the AI system matures, it is designed such that it can “adopt and challenge contradictory rules and behaviours” [43].

#### Risk of Harm

Patient safety concerns causing adverse effects were noted by Mlodzinski et al [48] and Vijayakumar et al [59]. The lack of accuracy of AI output was also considered to pose a potential risk of harm by both clinicians and patients, as, in some cases where the algorithm may output false negative results, it may provide an incorrect sense of reassurance and cause a delay in treatment. However, in cases where the algorithm is too sensitive, thereby providing false positive results, it may add work and costs to the treatment process [39,42,57,61,68]. The risk of harm can be lowered if AI algorithms are developed with the 5 rights (in the case of an automated decision system), similar to other clinical decision support tools [40]. In addition, Sangers et al [42] proposed that in some cases, AI applications should provide only risk indication instead of a diagnosis to reduce the risk of harm. Chen et al [68] reported that policies and mechanisms to safeguard professionals could address challenges associated with a potential risk of harm due to a lack of output accuracy.

#### Trust

Lack of accuracy; doubts about unsafe results; privacy breaches; patients’ perceptions and acceptance of automation; and uncertainty about developers’ reliability, availability, usability, and perceived usefulness were found to be obstacles to gaining trust [39,42,47,50,53,54,69,70]. Clinicians expressed fear around having to reframe their professional identity and responsibilities [57]. Fear around what AI really meant was noted as another barrier [36,42].

Facilitators of trust included the endorsement of the technology by experts as well as academically backed clinicians, including regulating bodies such as the government; evidence of output accuracy based on the evaluation of AI; and positive opinions from trusted thought leaders in the respective clinical fields [42,58,60]. Another facilitator of trust is the engagement of patients in the development of AI. This could facilitate trust in the public and address concerns around trust in data sharing [71]. Overall, trust was largely found to be associated with perceived usefulness; however, one study noted that “if peoples’ confidence and beliefs are improved, they will use the product despite its usefulness***”*** [52]. In addition, Fan et al [72] reported that initial trust is a key indicator of the intention to use the AI application and noted that if the confidence to use the AI application is high based on performance expectancy, then this will increase trust in using the AI.

#### Human-AI Teaming

The lack of human intervention was found to be a barrier to adoption for both clinicians and patients. From a clinician standpoint, physicians expressed that they would be less likely to use an AI, given their familiarity with the patient’s condition and the value of intuition in clinical decision-making [32,35]. Mlodzinski et al [48] reported concerns about potential systemic bias present in the AI that could impact the patient-provider relationship. From a patient standpoint, the lack of human presence was seen as a limitation due to a lack of empathy and emotional connectivity with another human or simply not having the presence of a human physician to verbally communicate and discuss, such as when using an AI app or chatbot [51,64]. By contrast, the lack of human presence was, in some cases, seen as a benefit due to the anonymity in sharing intimate or uncomfortable health concerns [39,42]. Hemphill et al [73] reported increased confidence in patients when AI was combined with clinical interpretation.

#### Aligning Strategic Components

Several studies particularly highlighted the importance of strategic alignment with initiatives. Baxter et al [35] reported on how a lack of alignment among different organizational initiatives led to varying outcomes and disjointed communication. Strohm et al [57], on the other hand, talked about how strategic alignment could lead to better dispersing of funds across different departments.

Sun and Medaglia [50] pointed out the necessity of outlining a comprehensive “top-down strategy” that would include organizations’ goals and resource distribution for AI implementation. Weinert et al [45] elaborated that to overcome the barrier to including AI initiatives in the organizational strategy, the German government introduced a new law that supported organizations with financial assistance to implement innovative digital technologies such as AI, as there was hesitancy among organizations to include expensive AI implementations as part of their strategy due to a lack of funds.

#### Use Case–Driven or Problem-Driven AI

Several studies noted that to start the journey of implementing AI, there is a need to identify a problem and not merely use data to come up with a solution. Therefore, use cases should be identified based on notable problems that can be addressed by AI solutions. One particular study mentioned how the lack of a use case affected the implementation of the AI model [35,36].

#### End-User Engagement or Co-Design

A lack of sufficient buy-in from end users and a lack of endorsement from organizational leadership emerged as barriers, including not engaging stakeholders early in the process. It is critical to incorporate clinicians and other stakeholders, such as patients, in the development life cycle, especially the testing phase with the application of a user-centered design and testing approaches. This may be time intensive but proves to be an effective approach to enable adoption [39,40]. As Ongena et al [69] pointed out in their findings, patients should be involved when developing AI systems, specifically for diagnostic, treatment planning, and prognostic purposes. Pou-Prom et al [74] reported the usefulness of engaging end users in designing, deploying, and refining the AI solution. Moorman [38] recommended maximizing buy-in and engagement at all levels of stakeholders, from leadership to users, and especially engaging a clinician leader from the onset. Finally, Goldstein et al [75] noted the inclusion of champions at the leadership and clinical levels to achieve successful implementation.

#### Workflow Integration

This review found that the lack of integration of the AI system into the workflow can be a barrier to successful implementation and adoption. For example, Baxter et al [35] reported impacts on success due to variations in existing workflows for risk assessments and readmission scores across different areas. Similarly, Strohm et al [57] mentioned how the lack of integration and standardization of workflows led to variations in workflows. For other types of AI solutions, such as apps, the data not being integrated into the health care system or workflow was seen as a barrier for patients to adopt the solution [42,53]. Helenason et al [58] and Schepart et al [56] both noted the importance of conformity with the workflow when integrating AI tools into the environment where they would be used.

Recommendations included the following: ensuring that AI applications easily integrate with existing IT systems, integrating data from patients’ use of AI-enabled apps into the health care system and workflow, and considering the integration of the AI into the clinical workflow but maintaining autonomy for clinicians to have the final say [19,20,38,42,49,57,75]. In situations where the AI system is deployed in different areas, using a common model may improve alignment in workflows [35,36]. Chen et al [68] reported that clinicians saw AI integration into the workflow as a positive if the system was seen as potentially eliminating routine work, allowing them to focus on other tasks. Moorman [38] reported that it was helpful to assess existing unit workflows, communications, escalation, and event management processes before implementation to address challenges brought up by clinicians concerned about added work.

#### Awareness and Training

Training refers to educating the users on various aspects of the technology, such as the outcomes of the AI model, its benefits, and how it supports the clinical workflow, and providing new skills such as technical and data science skills to staff, especially laggards and champions, to assist with the adoption [36,41,57,62]. Kelly et al [21] particularly noted that to provide clinicians with clarity on how an algorithm could improve patient care, approaches such as using a decision curve analysis that would provide quantified benefits of using a model to inform actions that need to be taken would be helpful. Skepticism and a lack of understanding were seen as barriers to AI adoption [48,51]. Chen et al [68] found that AI adoption increased among radiologists who were more familiar with AI. Sun [76] recommended that implementation teams should consider influencing clinicians by sharing AI knowledge via more informal communications, such as social media communication or in-person communication. Moorman [38] found it helpful to develop educational material with input from clinicians to tailor it to the clinical role and hospital culture. Training and awareness should include building an understanding of the technology; providing clarity around language such as the definition of AI; education around data use in health care; and building awareness on the value of AI, including breaking down concepts that dispel fear [36,37,39,42,50,52,71]. Clinicians’ awareness and knowledge of AI before using it contributed to its successful acceptance [59,73]. In addition, users feel that the technology is “unqualified” based on their perception of the premature nature of the technology [39]. Misunderstanding of the capabilities of AI technologies in the general public and the health care sector is a challenge to adoption, with gaps in awareness around the value, advantages, and high expectations of AI [37,50]. Overall, Alsobhi et al [70] emphasized the urgency of accelerating AI adoption through the dissemination of AI training.

#### Resources and Infrastructure

Shortages of personnel with the required skills were reported as barriers, along with the quality of IT infrastructure available for AI implementation [32,35,36,40,45,50]. Hickman et al [71], in particular, noted the lack of technological (infrastructure) maturity to allow for the integration of AI. The presence of AI experts, perhaps a multidisciplinary team, particularly with clinical scientists, data science and subject matter experts with AI skills, an innovation manager, AI experts to provide training, and local champions within departments involved in the end-to-end process, was considered an important element for adoption [32,57,59,62,71]. Goldstein et al [75] noted that for scalability, where the AI application would be deployed in multiple sites, having designated resources from the onset of the project with clear roles and responsibilities was seen with success. Yang et al [77] recommended cultivating talent with both high-level medical and technology knowledge and understanding how the 2 domains can be used to meet patients’ needs. In a different perspective on the shortage of resources, Chen et al [68] noted that radiographers and radiologists held more positive attitudes toward the adoption of AI, as it would help address workforce shortages in the radiology field in the United Kingdom.

#### Evaluation and Validation

The need for evaluation on multiple fronts was noted by various studies. Studies indicated that the technical evaluation of AI is necessary as a first step to validation. Technical evaluation must be followed by clinical validation (based on established methods in clinical research) and economic validation. Wolff et al [78] particularly noted the lack of clinical and economic measurements as a barrier to practical implementation. Evaluations should be tailored toward digital technologies to gather empirical evidence surrounding the value of AI’s use [53,78]. However, the cost of conducting an empirical evaluation and a quantified clinical trial–type evaluation may be a deterrent to the pace of developing the technology. Therefore, this should be considered when selecting the type of evaluation to be conducted. A focus on assessing the effectiveness and accuracy was duly highlighted. Implementers should consider validating or testing the algorithm with synthetic data [40-42,57]. Establishing a standard methodology for the validation of AI algorithms and the overall evaluation of AI will be critical to gaining confidence from adopters [50]. Hickman et al [71] suggested that having structures in place for the continued monitoring of standards that impact AI (eg, regulatory standards) and ensuring that infrastructure is in place to evaluate and monitor algorithms continuously are necessary.

#### Data Security, Ownership, Quality, and Availability

Data quality, security, ownership, and storage were prominent themes in the reviewed studies. In terms of data quality and integrity, several issues were identified as barriers to developing good AI models that provide value to users. These were issues around variability, the nature of unstructured data, incompleteness of data, the data not representing the reality of clinical care, and the absence of data standards (specifically around how and what data are collected). Having metadata standards, terminologies, data quality metrics, and common data models were identified as facilitators in resolving some of these issues [40,43,45,50]. Fragmented access to data and limited sources, such as the availability of data only from EHRs or data silos, were also noted as barriers [78].

Data access, integrity, and provenance are key to the development of models. Institutions that were the most successful in implementing AI were thoughtful about how to guarantee data integrity [40]. Wolff et al [78] noted the challenges with data silos and fragmented access to medical data, including limitations on the availability of data only from the EHR, to enable the development of robust AI applications. In terms of data ownership, the dilemma around who owns the data, whether it would be the government, institution (eg, hospital), or patient, is a barrier to adoption, as it leaves questions around how data would be integrated or accessible for future AI advancement [37,50]. While this may not present a direct adoption challenge, it does indirectly impact the availability of data required for development and to produce meaningful outputs, which is an impediment to adoption, as identified earlier.

Data security was identified as a major contributor toward hesitancy, with concerns around cybersecurity relating to both training and testing data as well as fear of trackers and spyware obtaining unsolicited data [39,41,42,45,48,77]. Furthermore, the ability of deidentified data to be reidentified poses a major risk for the individuals and institutions providing their data. This further contributes to resistance to sharing the data that could help expand data sets for AI training. Several strategies for preventing security breaches have been proposed, and these could be helpful in securing data, especially health data that are accessible over the web [20,42].

Concerns around data processing include the lack of understanding of how data are stored, processed, and accessed; the establishment of protocols; and compliance with existing privacy policies, such as the General Data Protection Regulation and the Health Insurance Portability and Accountability Act [20,36]. A survey of patients conducted by Ongena et al [69] indicated the need for patients to be informed about how and specifically which data are processed. One study explicitly highlighted the issue of data ownership. Concerns over data ownership were particularly evident when patients linked data ownership to trust in the technology [47].

#### Ethics and Privacy

Concerns about privacy and ethics were focused on maintaining confidentiality, ensuring processes are in place to obtain consent, and having informed consent with clarity on how the data will be processed [32,39,47,56]. Having clear consent processes related to how data are generated through the use of AI and how these data flow as well as defining the meaning of consent and transparency on strategies to maintain privacy are seen as facilitators of adoption. This is especially applicable to “clinical and epidemiological use cases of ML in both decision support and automation categories, as data from patients or the public are essential to train algorithms in these areas” [20]. In addition, Sun and Medaglia [50] particularly pointed out the unethical use of data, such as data being used by commercial organizations. Ensuring transparency to end users, especially patients involved in the ethical and legal frameworks that guide the development of AI systems, could be helpful [69,73]. Weinert et al [45] particularly identified ethical issues, specifically as they relate to liability, as a barrier to AI adoption. Wolff et al [78] noted that integrating “privacy-by-design” technologies into AI applications that incorporate advanced data protection features could mitigate such challenges.

#### Governance

Governance was primarily noted as a key facilitating factor, playing the role of enabling the full cycle of AI. It is critical to have a governance structure in place to oversee the development and rollout of AI from conception to implementation, with governance tools providing guidance on various stages of the process. Governance should include diverse professionals with clear articulation of accountability, including nuances in reactions to accountability [35,40,55,58,64]. Isbanner et al [64] noted the importance of articulating accountability. This is especially important in health care because “ethical and governance challenges matter to the public.” Wolff et al [78] recommended outlining specific responsibilities for different stakeholders to delineate accountability-based steps in the process, for example, identifying who would review an x-ray image analysis and identifying liability and culpability (eg, obligatory human check of a decision obtained by an AI application). According to Sunarti et al [47], the governance body should include “developers of software, government officials, health care, medical practitioners and advocacy for patients groups.” The lack of accountability in the decision-making process is a challenge; therefore, framing this in the governance model could be a way to address adoption issues related to accountability [37,50,73]. Other functions of the governance body would be to ensure funding and connectivity to the wider data science community, ensure alignment with strategic initiatives in the organization, and act as a long-term centralized knowledge repository for performance oversight. A governance model should have mechanisms and systems in place to facilitate changes impacting AI technologies in development or use based on cyclical changes in the technology or changes in the external landscape, such as the ones initiated by regulatory bodies [32,41,79]. Formalized analysis of ethical considerations in the development and use of AI should be a key component of governance. Governance should also be linked to the data governance committees for various data processing, quality, and integrity oversights [43]. One particular solution proposed by Morrison [36] was to have national-level governance templates that would facilitate national data protection via the implementation of impact assessments. In contrast, governance can hinder data sharing. Therefore, governance bodies should maintain a rigorous process without becoming a constraint [36].

#### Regulatory and Legal Frameworks

A lack of regulation and policies from the government, including uncertainty around legal direction or law, was presented as a barrier to the application of AI technologies [37,45,57,60,77,78]. Other researchers noted that there was no clarity in the area of regulatory structures with regard to which regulatory body should be consulted for AI developments and deployments [36,53]. Therefore, it would be essential for governments to establish regulatory bodies and legal frameworks to provide guidance on various aspects of AI development and application [20,41]. In addition, ambiguity surrounding malpractice liability policy as it relates to physicians’ legal responsibilities, for example, in case of diagnostic errors, remains a barrier to AI adoption [49].

#### Funding and Cost

The lack of and uncertainty surrounding funding are presented as barriers to implementation [32,51,56]. Researchers have suggested that there is a need to have costs identified from the start-up stage all the way to scalability. Funding can especially be a barrier if an AI technology is lacking in evidence, with little to show for the value it provides. Sun and Medaglia [50] and Xing et al [53] reported that financial barriers in the context of cost and benefits, the lack of a sustainable business model, and insufficient funding to meet public demands should also be considered. Sun and Medaglia [50] additionally noted challenges associated with the adoption of IBM Watson in China due to patients having to pay high fees for the service. Finally, funding should be cohesively considered not only for the development of the technology but also for resources required to implement the technology, such as technical subject matter experts, project managers, and champions [36,40,41,51,57]. Weinert et al [45] noted that to overcome the barrier of lack of resources and meet the financial investment demands of AI implementations, the German government introduced a new law that could help organizations bridge funding gaps; however, they could not conclude whether this would facilitate any progress, as the announcement was just made.

## Discussion

### Principal Findings

The principal findings of this study imply several factors impacting the adoption of AI systems, and for each barrier identified, there are corresponding facilitators. Ethics, bias, and transparency or explainability are core considerations in developing trustworthy and adoption-centric AI systems. Furthermore, the barriers identified should be holistically synergized within a governance framework, one that ideally oversees the entire end-to-end process, from ideation to the implementation and sustainability of AI systems.

Trust emerged as one of the most critical elements of AI adoption. This review revealed that trust can either be facilitated or impacted by almost all the themes identified in this scoping review. More specifically, fairness, explainability, and ethics seem to be the centerfold of barriers to AI adoption. Therefore, our discussions have focused on these 3 domains with recommendations on how organizations can address these domains to facilitate adoption.

### Transparency and Explainability

Our findings revealed that explainability in the context of algorithmic transparency is a significant barrier to adoption. Various studies noted that limitations due to the opacity of an algorithm may inhibit clinicians from relying on ML outputs in clinical settings. This leads to ambiguity on whether the ML output can be trusted enough for the clinician to move forward with the clinical decision-making or should be overridden due to a lack of certitude or misalignment with traditional clinical judgment [80]. AI explainability (XAI) is an entire field dedicated to ensuring trustworthy and explainable AI. There are numerous publications from this field. For example, Markus et al [81] noted that explanations are crucial to involving a human in the process of verifying the decision of the algorithm, for example, by revealing what features were used in training the AI algorithm. Adadi and Berrada [82] have conducted a comprehensive review of existing evidence on explainability approaches and organized them from different perspectives. They specifically outline 4 guidelines for the need for explainable AI. Explain to justify: the decisions made by using an underlying model should be explained to increase their justifiability. Explain to control: explanations should enhance the transparency of a model and its functioning, allowing its debugging and the identification of potential flaws. Explain to improve: explanations should help improve the accuracy and efficiency of their models. Explain to discover: explanations should support the extraction of novel knowledge and the learning of relationships and patterns to manage social interaction and create a shared meaning of the decision-making process.

There are several techniques that organizations can adopt when aiming to achieve explainable AI. These include explainable modeling, evaluating for explainability, or following an explainability framework, as proposed by Markus et al [81]. Preece [83] and Vilone and Longo [31] have done a thorough analysis of evaluation approaches for explainable AI. The inclusion of the combination of these techniques from XAI could be useful for organizations to address adoption barriers associated with explainability. It is prudent that organizations developing and implementing AI incorporate various explainable modeling approaches, include explainability frameworks, and consider explainability evaluation in their AI life cycle. In addition, part of this process should include equipping clinicians with knowledge about what the AI takes as input, how the input is processed, and what the AI produces as output, along with the training process used. In this way, clinician engagement is essential to the process of developing and validating AI algorithms and outputs. This approach will also empower clinicians to discuss these transparencies with patients, thereby contributing toward building trust on all fronts.

### Bias

In terms of equity and fairness, our findings have demonstrated that algorithm bias is a critical factor in not only gaining trust but also having meaningful outputs that can be applied to diverse patients. Specific concerns related to adoption include models being trained on data not representative of the patient population or not containing diverse data as related to social determinants of health [19,35,36]. Bias in AI systems can be introduced due to biased data, algorithms being trained on the biased data, limitations in the model itself, small training size, lack of user participation, and other unseen factors [84]. There are a number of examples of specific issues related to bias in AI systems. Buolamwini and Gebru [85] reported that an artificial vision algorithm was unable to identify dark-skinned individuals, as >80% of the individuals in a reference data set were light-skinned individuals. Another failed case is found in the field of anesthesiology, where data from 40 institutions revealed that Black patients received inferior care (with respect to postoperative nausea and vomiting prophylaxis) at nearly every single center [86]. Seyyed-Kalantari et al [87] noted that convolutional neural networks will frequently underdiagnose Hispanic patients at a disproportionate rate due to the potential lack of access to health care and insurance type. In the field of mental health, specifically concerning schizophrenia, a meta-analysis found that risk-flagging models trained on European populations have reduced performance in East Asian populations [88].

According to Panch et al [46], several challenges need to be addressed when addressing algorithmic bias. They include a lack of clear definitions and standards of “fairness,” insufficient contextual specificity, and the “black-box” nature of algorithms. These can be addressed by developing algorithms based on where they will be deployed by first establishing and identifying these contexts and ensuring processes are in place to address these challenges. There are numerous solutions to address bias that emphasize the risk of bias mitigation techniques to be applied at each stage of model development. For example, Chen et al [89] and O’Reilly-Shah [90] recommend that at the preprocessing stage, where the data may have internalized biases, techniques such as reweighing data samples of marginalized groups or resampling based on the population that algorithm output would be applied to. These techniques could help address the adoption barriers identified in this review, particularly those around underrepresentative and inaccurate training data sets. Similarly, at the postprocessing stage, similar thresholds for different representative groups could be set for the model to be monitored, adjusted, and trained. The design of the algorithm should consider equity via training data sets that are either diverse or focused to fit the localized population. Another major concern is data set drift, which means that the data set the model was trained with is different from the test data set. There are various techniques to mitigate data set shift, and these techniques should be considered in model development. Another mitigation technique, as recommended by Chen et al [89], is federated learning, where a model is trained on a global server. This technique allows for models to be trained on large data sets without sharing sensitive information. Aside from more quantitative techniques to address the risk of bias, there are assessments available that can be used as a checklist during each stage of AI development. While these tools can address statistical bias, it is much more challenging to identify social bias that can intrude into the data. Frameworks such as the one developed by Landers and Behrend [91] comprehensively outline questions that would be asked at each stage of AI development. These questions focus on information, perceptions, and other social and cultural components. Such tools, when integrated into the AI development process, would help gather evidence that could be shared with clinicians and patients on how bias mitigation has been considered in the end-to-end development process, thereby addressing adoption concerns around bias identified in this review.

### Ethics

Gerke et al [92] have discussed four primary ethical challenges that need to be addressed to realize the full potential of AI in health care: (1) informed consent to use, (2) safety and transparency, (3) algorithmic fairness and biases, and (4) data privacy. These challenges resonate with our findings around barriers to adoption and, interestingly, tie in these elements of barriers to adoption under the ethics domain.

There are several cases of ethical concerns that highlight the need for ethics. In the context of ethical concerns around informed consent and data privacy, in 2017, the personal data of approximately 1.6 million patients were provided to Google DeepMind by Royal Free National Health Service Foundation Trust without the patients’ consent. The data were to be used to test a new way of detecting kidney injuries [93]. From a clinician’s perspective, there are concerns around what the clinician’s responsibility is in informing patients about the use of AI in their care [92]. In the context of algorithmic safety and bias, Buolamwini and Gebru [85] and Liao [94] note ethical concerns around algorithms not detecting dark-skinned individuals for skin cancer detection due to the fact that the algorithm was trained on light-skinned individuals. Similarly, an algorithm that is widely used in US hospitals to identify patients who need additional care was found to use the cost expenditure by patients as a means to identify those who need extra care. This was discriminatory toward Black patients, as they generally spend less than White patients on health care, resulting in false conclusions [95].

In terms of safety and transparency, Liao [94] provides a good explanation as to why the lack of safety and transparency is an issue. They provide an example of a prediction of a 70% chance for a supposed patient’s tumor to become malignant in 5 years; however, the algorithm does not necessarily provide detailed reasons as to how it arrived at the conclusion. From an ethical standpoint, this is an issue because humans need to know how a decision is reached; specifically, in health care, not being able to understand and trust a decision is problematic.

Which and, more importantly, how can organizations address ethical concerns? According to Liao [94], there are >80 ethical frameworks that have been proposed for AI. Many of these draw on the 4 principles of biomedical ethics, namely, autonomy, beneficence, nonmaleficence, and justice. Some of these frameworks provide practical checklists that organizations can use to conduct an ethics deliberation. For example, Solanki et al [96] developed a comprehensive framework for AI developers that includes ethics oversight during different phases of the AI development life cycle. Similarly, Rogers et al [97] shared a very practical approach to how they evaluated an AI model for ethics. Such tools are practical methods of assessing AI algorithms for ethical principles. Despite these practical approaches, Goirand et al [98] note that operationalizing ethical frameworks for AI is challenging, as there is a need for contextualization due to different ethical issues present in different environments. Therefore, organizations have to consider these nuances and determine an ethics approach that would work best for their organization when evaluating each AI model.

**Figure 3:**
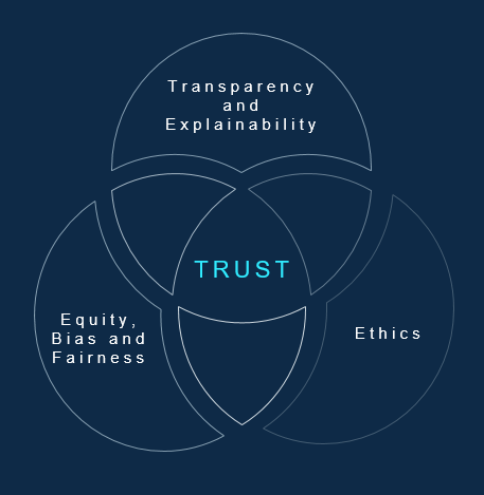
Adoption barriers, as related to trust.

These frameworks and tools to develop trustworthy AI by addressing various barriers to adoption are also just beginning to emerge and be applied in real-life cases; however, they are a good start to the implementation journey of AI, especially those applied in clinical settings. Overall, our findings demonstrate that the adoption of an AI system has to be considered from its onset, when the system is being conceptualized, to when it is implemented and sustained. The existing technology implementation and acceptance models may not be all encompassing of adoption factors; therefore, adding additional frameworks around trust, bias, explainability, and ethics will be necessary to foresee the success of an AI innovation. A governance model may address concerns around risk, safety, and adoption barriers identified in this paper by facilitating the overall development process of AI and ensuring various checks and balances are in place. Figure 3 is a visual depiction of the core elements that were found to impact trust, as discussed in this section.

### Limitations

Given the limited application of AI in health care at the time of this research, only a few number of papers that reported on implementation barriers and facilitators were reviewed to identify AI adoption barriers and facilitators. As the application of AI and types of AI systems in health care grows, a follow-up on adoption barriers and facilitators to assess for additional barriers and facilitators suitable to future environments may be necessary.

### Comparison With Prior Work

At the time this search was conducted, a few literature reviews on the determinants of and barriers to AI adoption were conducted, such as the review by Radhakrishnan and Chattopadhyay [26]. However, these studies span across multiple industries. For health care, one systematic review on the *barriers* to AI adoption in health care was conducted by Assadullah [27]. However, there is less work that considers both the *barriers to and facilitators* of AI adoption in health care at large. Therefore, this review has attempted to explore the latter to provide considerations for health care organizations looking to successfully implement AI technologies via increased adoption. Our findings are validated due to the replication of several themes identified in similar, previous research by Assadullah [27]. Common themes identified around barriers to adoption included explainability, trust issues centered on privacy, challenges around data ownership, lack of regulatory standards, issue of bias, and lack of accountability. Overall, the issue of trust was found to be centered on bias, ethics, and explainability, which led to a lack of accountability and an inability to evaluate. Other issues impeding trust included impacts on model performance leading to inaccurate results. These findings around trust resonate with results from this research, reinforcing the barriers to adoption identified in both studies.

### Conclusions

This literature review revealed that trust is impacted by a number of elements identified as barriers and that trust is a significant catalyst of adoption. A governance structure can be a key facilitator in ensuring all the elements identified as barriers are addressed appropriately. The findings demonstrate that the implementation of AI in health care is still in many ways dependent on the establishment of regulatory and legal frameworks. Further research around the combination of governance and implementation frameworks, models, or theories to enhance trust that would specifically enable adoption is needed to provide the necessary guidance to those translating AI research into practice. Future research could also be expanded to include attempts at understanding patients’ perspectives on complex, high-risk AI use cases and how the use of AI applications affect clinical practice and patient care, including sociotechnical considerations, as more algorithms are implemented in actual clinical environments.

## Appendix

Multimedia Appendix 1 PRISMA checklist.

## Abbreviations

AI: artificial intelligence
DL: deep learning
EHR: electronic health record
EMR: electronic medical record
ML: machine learning
NLP: natural language processing
PRISMA: Preferred Reporting Items for Systematic Reviews and Meta-Analyses
